# Analysing taxonomic structures and local ecological processes in temperate forests in North Eastern China

**DOI:** 10.1186/s12898-017-0143-y

**Published:** 2017-10-30

**Authors:** Chunyu Fan, Lingzhao Tan, Chunyu Zhang, Xiuhai Zhao, Klaus von Gadow

**Affiliations:** 1 Key Laboratory for Forest Resources & Ecosystem Processes of Beijing, Beijing Forestry University, Beijing, 100083 China; 2 Research Center of Forest Management Engineering of State Forestry Administration, Beijing Forestry Univerity, 100083 Beijing, China; 3 Department of Forest and Wood Science, University of Stellenbosch, Stellenbosch, South Africa; 4 Faculty of Forestry and Forest Ecology, Georg-August-University Göttingen, 37077 Göttingen, Germany

**Keywords:** Taxonomic structure, Environmental filtering, Dispersal limitation, Interspecific competition, Spatial scale, Temperate forest

## Abstract

**Background:**

One of the core issues of forest community ecology is the exploration of how ecological processes affect community structure. The relative importance of different processes is still under debate. This study addresses four questions: (1) how is the taxonomic structure of a forest community affected by spatial scale? (2) does the taxonomic structure reveal effects of local processes such as environmental filtering, dispersal limitation or interspecific competition at a local scale? (3) does the effect of local processes on the taxonomic structure vary with the spatial scale? (4) does the analysis based on taxonomic structures provide similar insights when compared with the use of phylogenetic information? Based on the data collected in two large forest observational field studies, the taxonomic structures of the plant communities were analyzed at different sampling scales using taxonomic ratios (number of genera/number of species, number of families/number of species), and the relationship between the number of higher taxa and the number of species. Two random null models were used and the “standardized effect size” (SES) of taxonomic ratios was calculated, to assess possible differences between the observed and simulated taxonomic structures, which may be caused by specific ecological processes. We further applied a phylogeny-based method to compare results with those of the taxonomic approach.

**Results:**

As expected, the taxonomic ratios decline with increasing grain size. The quantitative relationship between genera/families and species, described by a linearized power function, showed a good fit. With the exception of the family-species relationship in the Jiaohe study area, the exponents of the genus/family-species relationships did not show any scale dependent effects. The taxonomic ratios of the observed communities had significantly lower values than those of the simulated random community under the test of two null models at almost all scales. Null Model 2 which considered the spatial dispersion of species generated a taxonomic structure which proved to be more consistent with that in the observed community. As sampling sizes increased from 20 m × 20 m to 50 m × 50 m, the magnitudes of *SES*s of taxonomic ratios increased. Based on the phylogenetic analysis, we found that the Jiaohe plot was phylogenetically clustered at almost all scales. We detected significant phylogenetically overdispersion at the 20 m × 20 m and 30 m × 30 m scales in the Liangshui plot.

**Conclusions:**

The results suggest that the effect of abiotic filtering is greater than the effects of interspecific competition in shaping the local community at almost all scales. Local processes influence the taxonomic structures, but their combined effects vary with the spatial scale. The taxonomic approach provides similar insights as the phylogenetic approach, especially when we applied a more conservative null model. Analysing taxonomic structure may be a useful tool for communities where well-resolved phylogenetic data are not available.

**Electronic supplementary material:**

The online version of this article (doi:10.1186/s12898-017-0143-y) contains supplementary material, which is available to authorized users.

## Background

One of the core issues of forest community ecology is the identification of specific ecological processes that contribute to shaping community structure [1]. The assembly of a woody plant community in a forest may be regulated by various processes including regional history and local processes, such as abiotic and biotic interactions [2]. Local communities are built from a regionally available species pool. Within a given species pool, different ecological processes then shape community structure [3–6]. Specifically, more and more studies focus on the assessment of the relative importance of biotic and abiotic forces in community assembly [7].

Environmental filtering refers to abiotic factors that prevent the establishment or persistence of species in a particular location [8]. This concept involves the identification of particular species which are adapted to specific habitat conditions (such as terrain, soil or climate). According to the theory of niche conservation [9], species belonging to a particular genus or family could have similar ecological traits and habitat tolerance. Environmental filtering will therefore decrease the number of genera and families for a given number of species [9–11]. In contrast, interspecific competition may be especially intense between con-generic/familial species because of similar niche preferences. The similarity in the demand for resources may result in competitive exclusion, reducing the probability of coexistence of species from the same genus or family. Consequently, a “limiting similarity phenomenon” may be observed within a community [1, 12–14]. Thus, interspecific competition and environmental filtering have opposite impacts on the composition of different taxonomic levels. In addition to these niche-based ecological processes, dispersal limitation could also affect the species composition of local community [2, 5]. Through a spatial filtering effect, the species-genera/family ratios could increase.

Evolutionary relationships between species have been used to offer a new perspective for research regarding ecological processes [3–6, 15–17]. The ratios of generic or family richness to species richness (G/S and F/S, respectively), first used by Elton [18] in 55 animal and 27 plant communities in different habitats, present a simple and intuitive reflection of the “taxonomic structure” [19] of a community. Taxonomic structure could reflect the regulation of local processes, such as environmental filtering, interspecific competition and dispersal limitation by testing whether the co-occurring species are more closely related than would be expected by chance.

Many studies have used taxonomic structure to examine the effect of local processes quantitatively in real communities [2, 13, 15]. The construction of a taxonomic system for plants is mostly based on species’ phenotypic differences and similarities. Phenotypic variation has a basis in evolutionary history, and the taxonomic structure therefore contains information about genetic relationships among species to some extent [16]. While reviewing the historical debate on genus:species ratios, Jarvinen [20] noted the rarity of statistically robust empirical evidence for congeneric species coexisting less frequently than more distantly related taxa. A potential solution to this problem is to quantify the phylogenetic relatedness of co-occurring species.

The availability of phylogenies, along with methods for the construction of supertrees and for assembling the phylogenies of communities, now permits community structure to be assessed phylogenetically. Pairwise phylogenetic distances between species measure times of divergence during evolutionary history and are often argued to be a good synthetic measure of species ecological differentiation [15]. In a framework analogous to the taxonomic structure, the phylogenetic structure of communities can provide insights into the relative importance of different ecological processes. For example, if co-occurring species are more closely related than expected, i.e. phylogenetically clustered, this would be suggestive of abiotic filtering. Conversely, a phylogenetically overdispersed structure suggests that biotic interactions are more important in shaping a focal community [7, 21–25].

In line with similar studies in other regions, we try to understand the taxonomic characteristics of ecological communities, based on available information. Through a null modelling approach, the effect of local processes in shaping community assembly can be assessed by examining the deviations of the empirical patterns of taxonomic structure from null expectations [2, 18]. It is necessary to compare the results based on taxonomic structure with those based on phylogenetic data. We can thus test whether the conclusions about community assembly based on taxonomic structure are consistent. Moreover, phylogenetic analyses are being used extensively at global scales [16, 17], while the taxonomic structure is widely used to reflect underlying evolutionary principles of diversification along a wide environmental gradient. If the taxonomic structure reveals a pattern that is similar to the phylogenetic structure, it can be used more widely depending on the accessibility of data on species composition for taxa whose phylogenetic relationships are not well resolved.

The patterns and processes in a community change at different spatial scales [26]. When analysing different ecological processes, the sampling scale will affect the inferences. The scale effect thus requires special attention [27, 28]. For example, when the sample scale increases, interspecific competition will be weaker because of increasing resource availability at the larger areas [29]. As the effects of local processes vary with the grain size, the taxonomic and phylogenetic structure of a community may be scale-dependent as well [30].

Using data from very large (60-ha) observational field studies in two representative temperate forests in northeastern China, we will test four hypotheses: (1) the taxonomic structure of the two communities (i.e., taxonomic ratios, especially G/S and F/S and the exponents of genus/family-species relationships) are scale-dependent, (2) for a given species richness, ‘real’ communities consist of fewer numbers of genera and families than communities randomly assembled from a given species pool due to environmental filtering or dispersal limitation, suggesting that abiotic filtering is more important than interspecific competition in shaping a local community, (3) the effect of local processes on the taxonomic structure varies with the spatial scale, and (4) the analysis based on taxonomic structure provides similar insights when compared with the use of phylogenetic information.

## Materials and methods

### Study areas

The observations for this study were collected in two large forest plots located in Jiaohe, Jilin Province (east longitude 127°45′36.91″, north latitude of 43°58′05.60″) and Liangshui, Heilongjiang Province (east longitude 128°53′20″, north latitude 47°10′50″) in North-Eastern China. Both study areas, established in the summer of 2010, are located in a temperate continental mountain climate affected by monsoons. The study areas showed little human disturbance and represent a natural forest community.

The Jiaohe plot covers an area of 30 ha (500 m × 600 m), located within the administration of the Jilin Jiaohe Forestry Experimental Plot. The average temperature is − 18.6 °C during the coldest days in January, and 21.7 °C during the hottest days in July, with an average annual rainfall of 606 mm. The elevation ranges from 576 to 784 m above sea level, with fairly large topographic variation, mainly characterized by two slopes and a gully between. Slope directions are mainly southeasterly and southwesterly.

The Liangshui plot covers an area of 29.64 ha (380 m × 780 m), located in the Liangshui National Nature Reserve of Dailing District, Yichun City, Heilongjiang. The average temperature is − 6.6 °C during the coldest month and 7.5 °C during the hottest month. The annual average rainfall is 805 mm. The topography of the plot is flat with elevations ranging from 365 to 395 m.

Following the standard protocol for assessing large permanent field plots, all individual woody plants with a DBH ≥ 1 cm were recorded in the summer of 2010. All woody species (tree and shrub) encountered in the two observational study areas were identified. The scientific nomenclature followed the Flora of China (Additional file 1: Appendix 1). The Jiaohe plot contains 47 woody species, which belong to 30 genera of 18 families. The genus *Acer* includes most species: *A. barbinerve*, *A. mandshuricum*, *A. mono*, *A. ukurunduense*, *A. tegmentosum* and *A. triflorum*. Families with more than one species included *Aceraceae*, *Rosaceae* and *Betulaceae*. The Liangshui plot contained 31 woody species, belonging to 22 genera of 15 families. The genera with most species are *Picea*, *Populus* and *Acer*. The *Pinaceae* family is represented by five species, i.e., *Pinus koraiensis*, *Abies nephrolepis*, *Picea koraiensis*, *Picea jezoensis* and *Abies fabri*. In the Jiaohe study area, four topographic variables (slope, aspect, convexity and elevation) were assessed within 20 m × 20 m quadrats.

### Data analysis

#### Analysis of taxonomic structures

We divided each forest plot into a grid of cells (called quadrats in our study). In order to evaluate the sample scale dependence of the taxonomic structure, we considered five different quadrat sizes: 20 m × 20 m, 30 m × 30 m, 40 m × 40 m, 50 m × 50 m and 100 m × 100 m. The number of quadrats decreased as the quadrat size increased (details are presented in Additional file 1: Appendix 3). The ratios of the generic or family richness to species richness (G/S or F/S) were then calculated in each quadrat size. Several studies had shown that the taxonomic structure varied among habitats [31]. Therefore, the relationship between the taxonomic ratios and topographic variables were also examined using the Jiaohe observations in the 20 m × 20 m quadrats.

We further investigated the relationships between species richness and generic or family richness (species-higher taxon relationships). Previous studies have shown that both types of relationships can be adequately simulated by using the following models [2, 31]:

$$\ln \left( G \right) = a + b \times ln(S)$$

$$\ln \left( F \right) = a + b \times ln(S)$$

where *G* represents the number of genera, *F* the number of families and *S* the number of species. Because each species belongs only to one genus/family, the intercept parameter *a* was set to 0. Thus, the models were used in the following forms:

$$\ln \left( G \right) = b \times ln(S)$$

$$\ln \left( F \right) = b \times ln(S)$$

The exponent of the species-higher taxon relationship *b* can be estimated using regression analysis. The taxonomic ratios and exponents *b* provide a collective indicator of the taxonomic structure of communities.

#### Inferring local ecological processes from taxonomic structure

The null modelling approach was used to examine the influence of particular ecological processes by evaluating the deviations of the taxonomic structures between the observed and null communities. An appropriate null model should be chosen because in the forest, species are typically distributed non-randomly in space [32, 33]. Due to environmental factors and dispersal limitation of species, empirical communities share more species with nearby and ecologically similar communities than with distant and dissimilar ones. This positive spatial autocorrelation of species occurrence must therefore be considered in the null model. Otherwise, the probability that a null community is different from the empirical community would be high, thus increasing the type I error [16, 34].

*Null model 1* All species found in the study areas (the actual species pool) were considered to represent the local species pool. We assumed that each species had the same probability of occurring in any quadrat. Thus, in every quadrat of a particular spatial scale we held the species richness fixed at the observed value in the quadrat (preserving the column sums) and randomly selected species from the pool to build the corresponding null community model.

*Null model 2* Based on ecological realism, we sampled species for each quadrat in a probabilistic way considering the dispersion fields of species [16, 34, 35]. In the probabilistic framework, a species that occurs in several quadrats that share 10 species with the focal quadrat is more likely to be part of the focal quadrat’s source pool than a species that occurs in a quadrat that only shares a single species. This null model was constructed as follows: for an observed quadrat, we first sampled a quadrat from all quadrats with the same size weighted by the number of shared species, and then picked a species randomly from that quadrat. We then repeated this procedure until we obtained a quadrat with the number of species being equal with the observed one. The aim of using this similarity-weighted construction of a null community is to weaken the effects of both environmental filtering and dispersal limitation.

For the two null models, we calculated the number of genera and families in each null quadrat. To compare the empirical taxonomic structure with those from null models, we calculated a standardized effect size (SES) for each of the taxonomic ratios. This process was repeated 1000 times. The taxonomic ratios of the observed and null communities were compared to determine the dominant ecological processes affecting the taxonomic structure of each community. In the case of a strong abiotic effect in the community (e.g. environmental filtering or dispersal limitation), more congeneric/confamilial species would be expected to be present in the environment and the taxonomic ratios would be expected to be lower than those in the null hypothesis model. However, when the taxonomic structure was dominated by competition, due to the mutual exclusion of congeneric/confamilial species, the taxonomic ratios would be expected to be greater than those generated by the null hypothesis. That particular analysis is based on the, “standardized effect size” (*SES*) which is calculated as follows:

$${\text{SES = }}\frac{{I_{obs} - I_{null} }}{{\sigma_{null} }}$$

where *I*_*obs*_ indicates the observed taxonomic ratios in the actual community and $I_{null} \,{\text{and}}\, \sigma_{null}$ correspond to the mean and standard deviance of 1000 repeats of the null models, respectively. The variations in the *SES* values were analyzed simultaneously, for the different sample scales.

#### Phylogenetic structure test

Two phylogenetic supertrees were constructed for the species from each plot (Additional files 2, 3)  based on PhytoPhylo which was the updated version of the Zanne et al. [36] mega-phylogeny [37]. We estimated the commonly used nearest taxon index (NTI) which is a standardized measure of the phylogenetic distance to the nearest taxon (mean nearest taxon distance, MNTD) for each taxon in the sample. We computed NTI separately for each quadrat. The significance of NTI for an individual quadrat is assessed by comparing the observed MNTD with a null distribution of MNTD measured on 999 null communities. Null communities for a quadrat were created by randomly drawing an equal number of species from the plot-wide phylogeny. NTI then represents the standardized effect size (SES) of MNTD [38]. Positive values of NTI indicate that taxa are more related than expected (phylogenetically clustered), while negative values indicate that taxa are less related than expected (phylogenetically overdispersed).

NTI is calculated as:

$$NTI = - (MNTD_{obs} \,{-} \,mean (MNTD_{null} )) / sd(MNTD_{null} )$$

The MNTD_obs_ is the observed value of the mean nearest taxon distances. The mean (MNTD_null_) is the mean value from a null distribution where species names were randomly shuffled on the tips of the community phylogeny 999 times, and the MNTD values were calculated each time for each quadrat. The sd(MNTD_null_) is the standard deviation of the null distribution. For a more intuitive and convenient comparison between the results of taxonomic and phylogenetic structures, we used − 1 × NTI in our study.

A Student’s t test was used to test for significant deviations of NTI from the expectation of zero. To test whether the phylogenetic structure of local communities depends on the spatial scale, an ANOVA was performed to detect differences among NTI at different scales. A similar test was also applied to SES of the taxonomic ratios.

All statistical analyses were conducted with the software R 3.3.3 (R Development Core Team).

## Results

### Taxonomic structures

The ratios of generic richness to species richness (G/S) were 0.64 in the Jiaohe and 0.71 in the Liangshui study areas. The ratios of family richness to species richness (F/S) were 0.38 and 0.48, respectively.

The G/S and F/S ratios were related to the spatial scales. With increasing area, the value of both ratios, as the mean of all quadrats, decreased in both study areas (Table 1). Based on the analysis of the environmental data of Jiaohe on the 20 m × 20 m scale, we found that of the four major terrain factors, the variables with significant correlation to the ratio of generic richness to species richness were elevation, aspect, and convexity. The ratio of family richness to species richness showed a significant correlation with elevation (Table 2).

**Table 1:** Ratios of generic richness to species richness (G/S) and of family richness to species richness (F/S) at five different spatial scales

Research plot	Spatial scale	Genus/species (G/S)			Family/species (F/S)		
		Max	Min	Mean	Max	Min	Mean
Jiaohe	20 m × 20 m	1.00	0.54	*0.76*	1.00	0.36	*0.63*
	30 m × 30 m	0.91	0.6	*0.75*	0.75	0.41	*0.57*
	40 m × 40 m	0.86	0.59	*0.74*	0.73	0.40	*0.55*
	50 m × 50 m	0.83	0.64	*0.73*	0.63	0.40	*0.52*
	100 m × 100 m	0.79	0.62	*0.7*	0.55	0.43	*0.48*
Liangshui	20 m × 20 m	1.00	0.55	*0.82*	1.00	0.33	*0.58*
	30 m × 30 m	1.00	0.63	*0.78*	0.75	0.35	*0.51*
	40 m × 40 m	0.91	0.67	*0.76*	0.63	0.35	*0.49*
	50 m × 50 m	0.83	0.67	*0.75*	0.59	0.38	*0.47*
	100 m × 100 m	0.78	0.69	*0.74*	0.57	0.41	*0.47*

**Table 2:** Pearson correlation coefficients and their confidence intervals (in brackets) between the taxonomic ratio (G/S or F/S) and topographic variables at the 20 m × 20 m scale in Jiaohe

Topographic variable	G/S	F/S
Elevation	− 0.11** (− 0.18, − 0.04)	− 0.11* (− 0.18, − 0.04)
Slope	− 0.06 (− 0.13, 0.02)	− 0.01 (− 0.08, 0.06)
Aspect	− 0.12*** (0.06, 0.20)	− 0.07 (− 0.01, 0.14)
Convexity	− 0.09* (0.02, 0.16)	− 0.04 (− 0.11, 0.04)

*** Indicates p < 0.001, ** indicates p < 0.01, * indicates p < 0.05

**Fig. 1:**
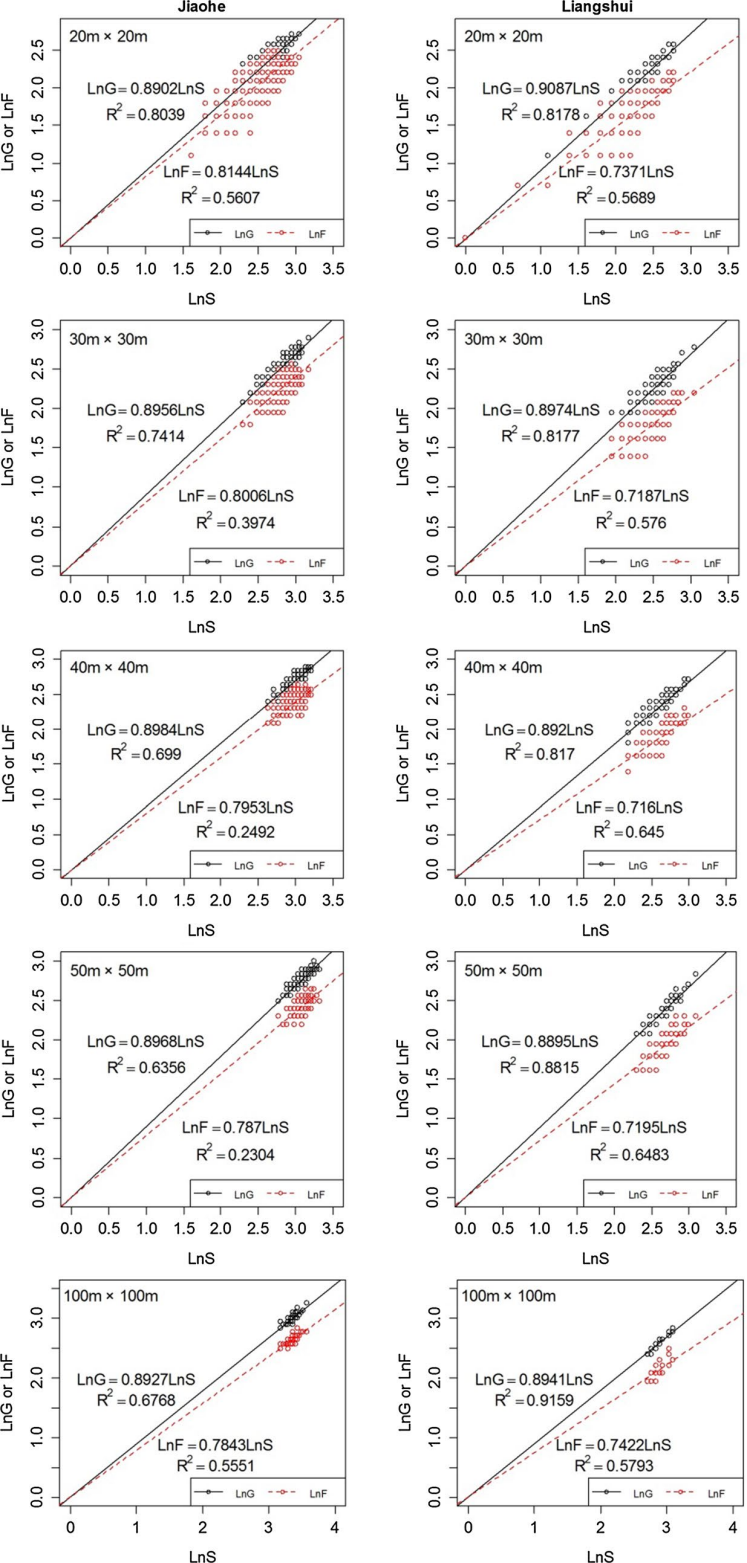
The relationships between species richness and generic/family richness (black line/red dashed line) in the Jiaohe and Liangshui study areas

**Fig. 2:**
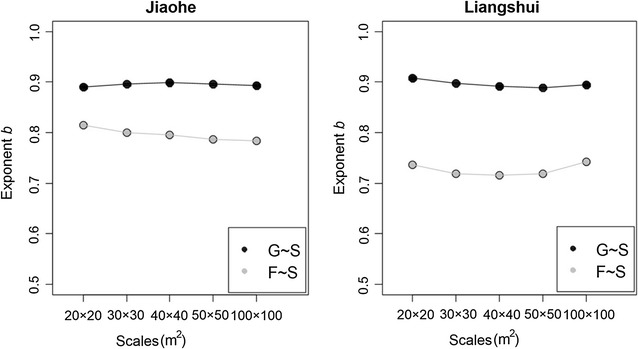
Exponents of genus-species and family-species relationships in the Jiaohe and Liangshui study areas at different sample scales

We used a power function to estimate the relationship between species richness and generic/family richness across quadrats (Fig. 1). As for the genus-species relationship, the exponents, determined on all five scales in Liangshui (Fig. 2), showing little variation. In comparison to the genus-species relationship, the family-species relationship in both study areas displayed greater stability with the change of scale. For example, the exponent in Jiaohe decreased with successive increases in scale.

### Taxonomy-based test

**Fig. 3:**
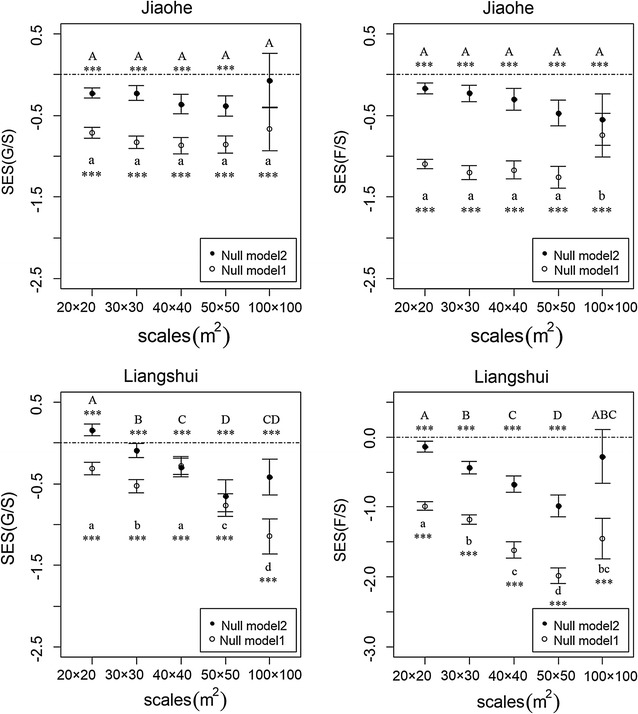
The standardized effect size (*SES*) (mean value and the 95% confidence interval) of the two null models at different scales in the Jiaohe and Liangshui study areas. Notes: Different capital letters indicate significant differences among different spatial scales under Null model 2, *** indicate that *SES* of taxonomic ratios at a given scale differs from 0. Different lowercase letters indicate significant differences among different spatial scales under Null model 1, *** indicate that *SES* of taxonomic ratios at a given scale differs from 0

For the Jiaohe plot, the SESs of genus—(G/S) and family to species (F/S) ratios were negative or not significantly different from 0. As sampling sizes increased from 20 m × 20 m to 50 m × 50 m, the magnitudes of SESs of taxonomic ratios also increased. We detected a positive mean SES of genus to species ratio (G/S) for the Liangshui plot at the 20 m × 20 m scale. The deviation of taxonomic structure between the empirical and simulated community decreased under Null model 2, relative to Null model 1 (Fig. 3).

### Phylogeny-based test

**Fig. 4:**
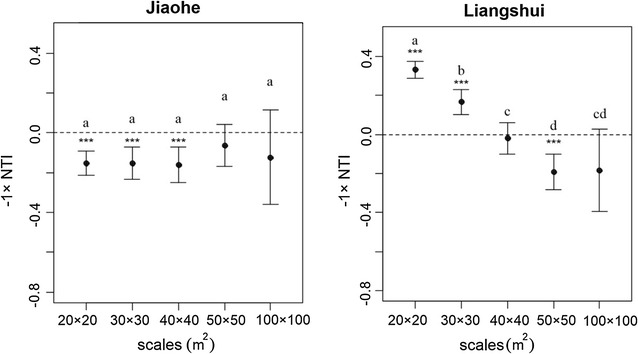
The − 1 × NTI distributions (mean value and the 95% confidence interval) under different spatial scales. A positive value means phylogenetic overdispersion; a negative value means clustered. Notes: Different lowercase letters indicate significant differences among different spatial scales, *** indicates that the phylogenetic structure at a given scale differs from 0

The Jiaohe plot was phylogenetically clustered at the scales from 20 m × 20 m to 40 m × 40 m, with NTI significantly greater than 0. Although the mean NTI > 0 at the scales of 50 m × 50 m and 100 m × 100 m, indicating a slight overall trend of phylogenetic clustering at the two scales, these quadrats were not phylogenetically clustered or overdispersed because NTI did not differ significantly from 0. The phylogenetic structure in the Jiaohe plot showed no scale dependency. We detected significant phylogenetic overdispersion at the 20 m × 20 m and 30 m × 30 m scales in the Liangshui study area. As the scale increased, the phylogenetic structure became clustered, with positive values of NTI (Fig. 4).

## Discussion

### Taxonomic structure of the two communities

Enquist et al. [2] used data from woody plant communities in different biogeographic regions, continents and geologic time periods to identify that there was a general pattern in the taxonomic structure and found that the genus/family-species relationship could be effectively described by a power function. This type of analysis has been applied to communities of animals, plants and microbes [2, 11, 18]. Our results, consistent with previous studies, showed that model fit was satisfactory and the taxonomic structure of forest community presented a pattern that is similar with other types of communities [31]. As the number of species increases, the number of genera/families was also increasing. The taxonomic structure represents the rate of diversification of the genus or family, relative to the level of the species [15, 31, 39].

The taxonomic ratios of Jiaohe showed a significant relationship with topographic variables. These results suggest that differences in the taxonomic structure may significantly differ among environments. An increase in species richness was mainly attributable to species that belonged to the same higher taxon. For a given genus richness, niche differentiation was greater at higher elevations. This result indicates an environmental constraint affecting the taxonomic composition of forest communities in Jiaohe [31].

### Local ecological processes

In this study, we applied a phylogenetic approach to detect community assembly processes and compared the results to those obtained with a taxonomic approach. The general trends were very similar between the two methods. We found phylogenetic clustering in the Jiaohe plot at almost all scales and phylogenetic overdispersion at fine scales in the Liangshui study area.

The taxonomic ratios scaling exponents of the genus/family-species relationships in the observed communities were found to be significantly lower than those in the two simulated null communities at almost all scales. This shows that for a given species richness, our observed communities have fewer numbers of genera and families than random communities based on the studied species pools. These results suggest that abiotic filtering was more effective in determining the current taxonomic structures, which confirms earlier investigations [13, 40, 41]. Swenson et al. [7] found that the effect of competition could significantly change the phylogenetic structure of a tropical forest community, but only at scales less than 5 m × 5 m. The effect of environmental filtering was always a dominant factor at greater scales. Wang et al. [18] reached the same conclusion based on their research in temperate forest communities in China, where the effect of abiotic filtering was always greater than the effect of competition.

In the Liangshui plot, we found that the mean *SES* of G/S was positive and phylogenetically overdispersed at fine scales, indicating intense competitive exclusion. The communities at these scales in the Liangshui plot mainly consisted of species from two speciose lineages, *Pinus* and *Acer. Pinus* and *Acer* have many congeners and thus may be more likely to show overdispersion than less species lineages if, for example, increased diversity leads to increased competition among closely related species.

In developing Null Model 2 for a taxonomy-based test, rather than arbitrarily choosing a species, we considered the probability of a species occurring in a specific simulated quadrat, thus accounting for the effects of environmental filtering and dispersal limitation to some degree. As the null model is restricted, the deviation of the empirical taxonomic structure from null expectation decreased, suggesting reduced regulatory effects caused by environmental filtering or dispersal limitation. Compared to Null Model 1, Null Model 2 thus generated a taxonomic structure that was more consistent with the empirical one. These results further confirmed the dominant influence of environmental filtering and dispersal limitation. It was necessary to preserve the spatial dispersion of species to avoid making an arbitrary inference of the effect of a particular process [34].

Recently, many studies have shown that the effects of environmental filtering have been largely overestimated [8]. Mayfield and Levine argued that interspecific competition would only occasionally eliminate more closely related species and that competition exclusion caused by the fitness difference between species will result in phylogenetic clustering [17]. For example, in a hypothetical light-limited environment, a fitness difference between species may be indicated by the height of individuals, which may be indicative of a competitive ability difference. Competitive exclusion will preferentially eliminate species with slow height growth, which may cause more distantly related competitors less likely to coexist. We found some evidence of environmental filtering or dispersal limitation which may also reflect the influence of competitive exclusion resulting from competitive advantages like tree height to some extent. The role of competition in shaping community assemblies requires more attention in future studies. However, this is not a trivial problem which requires assessment of multiple competition effects (root competition, crowding and overtopping) and requires analysis of multiple response patterns for different species, tree dimensions and development stages, as has been shown by Seifert et al. [42].

### Scale dependence

We found a clear downward trend in the taxonomic ratios with increasing spatial scale. This phenomenon seems to be closely related to changes in the intensity of different local ecological processes as the spatial scale increases. As the sampled area increases, more essential resources become available and competition between species with similar resource requirements is reduced [26, 29]. Hence, more congeneric/confamilial species are found on larger plots. However, any increase in environmental heterogeneity and space weakens the intensity of the effects of environmental filtering [9, 43, 44], thus increasing the probability of species belonging to various genera and families within a given community [19]. It is thus difficult to distinguish between the effects of environmental filtering/dispersal limitation and interspecific competition. However, in this study we found evidence of abiotic filtering (e.g. environmental filtering and dispersal limitation) at almost all scales. Consequently, we conclude that variations in the taxonomic structure with increasing scale of the subsample are due to the reduced effects of interspecific competition, which increases the probability of co-existence of congeneric/confamilial species in the local community [11, 45]. The scale dependence of the taxonomic structure is the result of the combined effect of the two types of local processes.

It appears that, as the scale increases, the magnitude of *SES* of genus—(G/S) and family (F/S) to species ratios, which reflects the combined effects of abiotic filtering and interspecific competition, increases from the 20 m × 20 m to the 50 m × 50 m quadrat size, suggesting that the species composition of observed communities became more closely related. At greater scales, the observed taxonomic structure is more similar to that found in a random assemblage community which further suggests that the balance effect of opposing processes are changing along spatial scales [22].

Surprisingly, the analyses of scale-dependent taxonomic structures provided similar insights when compared with the results of the phylogenetic analyses, especially when we applied a more conservative null model. The spatial scaling results for Jiaohe using phylogenetic methods are consistent with those of a recent study by Kembel and Hubbell [40]. This study found a clustered to random signal across spatial scales ranging from 400 m^2^ to 1 ha. The random or close-to-random structure observed at larger scales and the lack of significant NTI clustering at larger scales could be due to lower power. Other recent work using phylogenies found that at spatial scales finer than 100 m^2^, phylogenetic overdispersion is more evident [28], similar to our result in Liangshui. At larger spatial scales, the overdispersed structure progressively turns into a random or clustered structure. This suggests that the degree of phylogenetic relatedness between co-occurring species is most important at very small and very large spatial scales. It is still unclear whether the random pattern detected at the 50 m × 50 m scale in our study is due to the mixing of overdispersion and clustering or is actually indicative of neutral processes.

## Conclusions

The analysis of taxonomic structures provides insights that are similar to those obtained using phylogenetic information, especially when a conservative null model is applied. The effect of environmental filtering and dispersal limitation in our temperate forest community was found to be greater than the effect of interspecific competition in shaping the local tree community at almost all scales. This result is based on both, the taxonomic and the phylogenetic structure. Local processes do influence the taxonomic structure, but their combined effects may vary with scale. The taxonomic and phylogenetic approaches used in this study can help to explain the particular assembly of the temperate forest community. The phylogenetic structure was influenced by the accuracy of the phylogeny, the grouping into tree size classes and the chosen phylogenetic index. For improved understanding of variations in community structure at different spatial scales, we suggest that in future studies information on species functional traits need to be included.

## Additional files

**Additional file 1.** Species, genera and families in Liangshui study area; species, genera and families in Jiaohe study area; numbers of quadrats at five different scales in Jiaohe and Liangshui study areas.

**Additional file 2.** The phylogenies for Jiaohe plot.

**Additional file 3.** The phylogenies for Liangshui plot.

## References

[1] Tilman D (2004). Niche tradeoffs, neutrality, and community structure: a stochastic theory of resource competition, invasion, and community assembly. P Natl Acad Sci..

[2] Enquist BJ, Haskell JP, Tiffney BH (2002). General patterns of taxonomic and biomass partitioning in extant and fossil plant communities. Nature.

[3] Zobel M (1992). Plant species co-existence: the role of historical, evolutionary and ecological factors. Oikos.

[4] Eriksson O (1993). The species-pool hypothesis and plant community diversity. Oikos.

[5] Vellend M (2010). Conceptual synthesis in community ecology. Q Rev Biol..

[6] Ernest SK (2008). Zero sum, the niche, and meta-communities: long-term dynamics of community assembly. Am Nat.

[7] Swenson NG, Enquist BJ, Jill T, Zimmerman JK (2007). The influence of spatial and size scale on phylogenetic relatedness in tropical forest communities. Ecology.

[8] Kraft NJB, Adler PB, Godoy O, James EC, Fuller S, Levine JM (2014). Community assembly, coexistence and the environmental filtering metaphor. Funct Ecol.

[9] Jabot F, Chave J (2011). Analyzing tropical forest tree species abundance distributions using a nonneutral model and through approximate bayesian inference. Am Nat.

[10] Lavorel S, Garnier E (2002). Predicting changes in community composition and ecosystem functioning from plant traits: revisiting the holy grail. Funct Ecol.

[11] Werner U (2010). Species assortment or habitat filtering: a case study of spider communities on lake islands. Ecol Res.

[12] Kraft NJB, Valencia R, Ackerly DD (2008). Functional traits and niche-based tree community assembly in an Amazonian Forest. Science.

[13] Claire AB (2013). A taxonomic comparison of local habitat niches of tropical trees. Oecologia.

[14] Hubbell SP (2001). The unified neutral theory of biodiversity and biogeography.

[15] Mayfield MM, Levine JM (2010). Opposing effects of competitive exclusion on the phylogenetic structure of communities. Ecol Lett.

[16] Wang S (2012). The influence of species pools and local processes on the community structure: a test case with woody plant communities in China’s mountains. Ecography.

[17] Gómez JP (2010). A phylogenetic approach to disentangling the role of competition and habitat filtering in community assembly of Neotropical forest birds. J Anim Ecol.

[18] Elton C (1946). Competition and the structure of ecological communities. J Anim Ecol.

[19] Lessard JP (2012). Inferring local ecological processes amid species pool influences. Trends Ecol Evol.

[20] Jarvinen O (1982). Species-to-genus ratios in biogeography: a historical note. J Biogeogr.

[21] Webb CO, Ackerly DD, Mcpeek MA, Donoghue MJ (2002). Phylogenies and community ecology. Annu Rev Ecol Evol S..

[22] Zobel M (2011). The formation of species pools: historical habitat abundance affects current local diversity. Global Ecol Biogeogr..

[23] Lessard JP (2015). Process-based species pools reveal the hidden signature of biotic interactions amid the influence of temperature filtering. Am Nat.

[24] Montaña CG, Winemiller KO, Sutton A (2014). Intercontinental comparison of fish ecomorphology: null model tests of community assembly at the patch scale in rivers. Ecol Monog..

[25] Zanne AE, Tank DC, Cornwell WK (2013). Corrigendum: three keys to the radiation of angiosperms into freezing environments. Nature.

[26] Rahbek C (2005). The role of spatial scale and the perception of large-scale species-richness patterns. Ecol Lett.

[27] Zhang C (2014). Scale dependent structuring of spatial diversity in two temperate forest communities. Forest Ecol Manag..

[28] Cavender BJ, Adrienne K, Brianna M (2006). Phylogenetic structure of Floridian plant communities depends on taxonomic and spatial scale. Ecology.

[29] Weiher E (2011). Advances, challenges and a developing synthesis of ecological community assembly theory. Philos T R Soc B..

[30] Chase JM, Knight TM (2013). Scale-dependent effect sizes of ecological drivers on biodiversity: why standardised sampling is not enough. Ecol Lett.

[31] Passy S, Legendre P (2006). Power law relationships among hierarchical taxonomic categories in algae reveal a new paradox of the plankton. Global Ecol Biogeog..

[32] Roxburgh SH, Chesson P (1998). A new method for detecting species associations with spatially autocorrelated data. Ecology.

[33] Palmer MW, van der Maarel E (1995). Variance in species richness, species association, and niche limitation. Oikos..

[34] Gotelli NJ (2000). Null model analysis of species co-occurrence patterns. Ecology.

[35] Burns JH, Strauss SY (2011). More closely related species are more ecologically similar in an experimental test. P Natl Acad Sci USA.

[36] Zanne AE (2014). Three keys to the radiation of angiosperms into freezing environments. Nature.

[37] Qian H, Jin Y (2016). An updated megaphylogeny of plants, a tool for generating plant phylogenies and an analysis of phylogenetic community structure. J Plant Ecol..

[38] Kraft NJB, Cornwell WK, Webb CO, Ackerly DD (2007). Trait evolution, community assembly, and the phylogenetic structure of ecological communities. Am Nat.

[39] Mouillot D, Poulin R (2004). Taxonomic partitioning shedding light on the diversification of parasite communities. Oikos.

[40] Kembel SW, Hubbell SP (2006). The phylogenetic structure of a neotropical forest tree community. Ecology.

[41] Wang X (2013). Phylogenetic and functional diversity area relationships in two temperate forests. Ecography.

[42] Seifert T, Seifert S, Seydack A, Durrheim G, Gadow K (2014). Competition effects in an Afrotemperate Forest. For Ecosyst.

[43] Lessard JP, Sanders NJ (2012). Strong influence of regional species pools on continent-wide structuring of local communities. P Roy Soc B Biol Sci..

[44] Zhang C (2012). Species-habitat associations in a northern temperate forest in China. Silva Fenn..

[45] Stokes CJ, Archer SR (2010). Niche differentiation and neutral theory: an integrated perspective on shrub assemblages in a parkland savanna. Ecology.

