# Supplementary material for: Analysing taxonomic structures and local ecological processes in temperate forests in North Eastern China
Source: BMC Ecol. 2017 Oct 30;17:33. doi: 10.1186/s12898-017-0143-y (PMC5663035; doi:10.1186/s12898-017-0143-y)
Supplement: Supplementary file 1 — Additional file 1. Species, genera and families in Liangshui study area; species, genera and families in Jiaohe study area; numbers of quadrats at five different scales in Jiaohe and Liangshui study areas. [file 12898_2017_143_MOESM1_ESM.docx]

**Appendix**

**Appendix 1** Species, genera and families in Liangshui study area.

| Species | Family | Genus |
| --- | --- | --- |
| *Maackia amurensis* | *Leguminosae* | *Maackia* |
| *Tilia amurensis* | *Tiliaceae* | *Tilia* |
| *Juglans mandshurica* | *Juglandaceae* | *Juglans* |
| *Corylus mandshurica* | *Betulaceae* | *Corylus* |
| *Betula costata* | *Betulaceae* | *Betula* |
| *Betula platyphylla* | *Betulaceae* | *Betula* |
| *Quercus mongolica* | *Fagaceae* | *Quercus* |
| *Syringa reticulata* | *Oleaceae* | *Syringa* |
| *Fraxinus mandshurica* | *Oleaceae* | *Fraxinus* |
| *Acer mono* | *Aceraceae* | *Acer* |
| *Acer ukurunduense* | *Aceraceae* | *Acer* |
| *Acer tegmentosum* | *Aceraceae* | *Acer* |
| *Padus racemose* | *Rosaceae* | *Padus* |
| *Lonicera maackii* | *Caprifoliaceae* | *Lonicera* |
| *Rhamnus davurica* | *Rhamnaceae* | *Rhamnus* |
| *Pinus koraiensis* | *Pinaceae* | *Pinus* |
| *Eleutherococcus senticosus* | *Araliaceae* | *Acanthopanax* |
| *Populus davidiana* | *Salicaceae* | *Populus* |
| *Ulmus japonica* | *Ulmaceae* | *Ulmus* |
| *Phellodendron amurense* | *Rutaceae* | *Phellodendron* |
| *Abies nephrolepis* | *Pinaceae* | *Abies* |
| *Populus cathayana* | *Salicaceae* | *Populus* |
| *Salix matsudana* | *Salicaceae* | *Salix* |
| *Populus nigra* | *Salicaceae* | *Populus* |
| *Picea koraiensis* | *Pinaceae* | *Picea* |
| *Rhamnus diamantiaca* | *Rhamnaceae* | *Rhamnus* |
| *Abies fabri* | *Pinaceae* | *Abies* |
| *Alnus sibirica* | *Betulaceae* | *Alnus* |
| *Amygdalus davidiana* | *Rosaceae* | *Amygdalus* |
| *Picea jezoensis* | *Pinaceae* | *Picea* |
| *Picea asperata* | *Pinaceae* | *Picea* |

**Appendix 2** Species, genera and families in Jiaohe study area.

| Species | Family | Genus |
| --- | --- | --- |
| *Maackia amurensis* | *Leguminosae* | *Maackia* |
| *Tilia amurensis* | *Tiliaceae* | *Tilia* |
| *Tilia mandshurica* | *Tiliaceae* | *Tilia* |
| *Juglans mandshurica* | *Juglandaceae* | *Juglans* |
| *Philadelphus schrenkii* | *Saxifragaceae* | *Philadelphus* |
| *Deutzia parviflora* | *Saxifragaceae* | *Philadelphus* |
| *Carpinus cordata* | *Betulaceae* | *Carpinus* |
| *Corylus mandshurica* | *Betulaceae* | *Corylus* |
| *Betula costata* | *Betulaceae* | *Betula* |
| *Betula dahurica* | *Betulaceae* | *Betula* |
| *Betula platyphylla* | *Betulaceae* | *Betula* |
| *Quercus mongolica* | *Fagaceae* | *Quercus* |
| *Syringa reticulata* | *Oleaceae* | *Syringa* |
| *Fraxinus mandshurica* | *Oleaceae* | *Fraxinus* |
| *Fraxinus rhynchophylla* | *Oleaceae* | *Fraxinus* |
| *Vitis amurensis* | *Vitaceae* | *Vitis* |
| *Acer barbinerve* | *Aceraceae* | *Acer* |
| *Acer mandshuricum* | *Aceraceae* | *Acer* |
| *Acer mono* | *Aceraceae* | *Acer* |
| *Acer ukurunduense* | *Aceraceae* | *Acer* |
| *Acer tegmentosum* | *Aceraceae* | *Acer* |
| *Acer triflorum* | *Aceraceae* | *Acer* |
| *Sorbus alnifolia* | *Rosaceae* | *Sorbus* |
| *Padus racemose* | *Rosaceae* | *Padus* |
| *Cerasus maximowiczii* | *Rosaceae* | *Prunus* |
| *Sorbus pohuashanensis* | *Rosaceae* | *Sorbus* |
| *Malus baccata* | *Rosaceae* | *Malus* |
| *Crataegus maximowiczii* | *Rosaceae* | *Crataegus* |
| *Lonicera maackii* | *Caprifoliaceae* | *Lonicera* |
| *Lonicera chrysantha* | *Caprifoliaceae* | *Lonicera* |
| *Lonicera ruprechtiana* | *Caprifoliaceae* | *Lonicera* |
| *Sambucus williamsii* | *Caprifoliaceae* | *Sambucus* |
| *Rhamnus davurica* | *Rhamnaceae* | *Rhamnus* |
| *Rhamnus schneideri* | *Rhamnaceae* | *Rhamnus* |
| *Pinus koraiensis* | *Pinaceae* | *Pinus* |
| *Abies holophylla* | *Pinaceae* | *Abies* |
| *Euonymus phellomana* | *Celastraceae* | *Euonymus* |
| *Euonymus verrucosus* | *Celastraceae* | *Euonymus* |
| *Eleutherococcus senticosus* | *Araliaceae* | *Acanthopanax* |
| *Aralia elata* | *Araliaceae* | *Aralia* |
| *Populus koreana* | *Salicaceae* | *Populus* |
| *Populus davidiana* | *Salicaceae* | *Populus* |
| *Salix koreensis* | *Salicaceae* | *Salix* |
| *Ulmus laciniata* | *Ulmaceae* | *Ulmus* |
| *Ulmus macrocarpa* | *Ulmaceae* | *Ulmus* |
| *Ulmus japonica* | *Ulmaceae* | *Ulmus* |
| *Phellodendron amurense* | *Rutaceae* | *Phellodendron* |

**Appendix 3** Numbers of quadrats at five different scales in Jiaohe and Liangshui study areas.

| Research plot | Sampling size | Number of samples |
| --- | --- | --- |
|  |  |  |
| Jiaohe | 20m×20m | 750 |
|  | 30m×30m | 320 |
|  | 40m×40m | 180 |
|  | 50m×50m | 120 |
|  | 100m×100m | 30 |
| Liangshui | 20m×20m | 740 |
|  | 30m×30m | 312 |
|  | 40m×40m | 171 |
|  | 50m×50m | 105 |
|  | 100m×100m | 21 |
