# Supplementary figures and images for: Analysing taxonomic structures and local ecological processes in temperate forests in North Eastern China
Source: BMC Ecol. 2017 Oct 30;17:33. doi: 10.1186/s12898-017-0143-y (PMC5663035; doi:10.1186/s12898-017-0143-y)

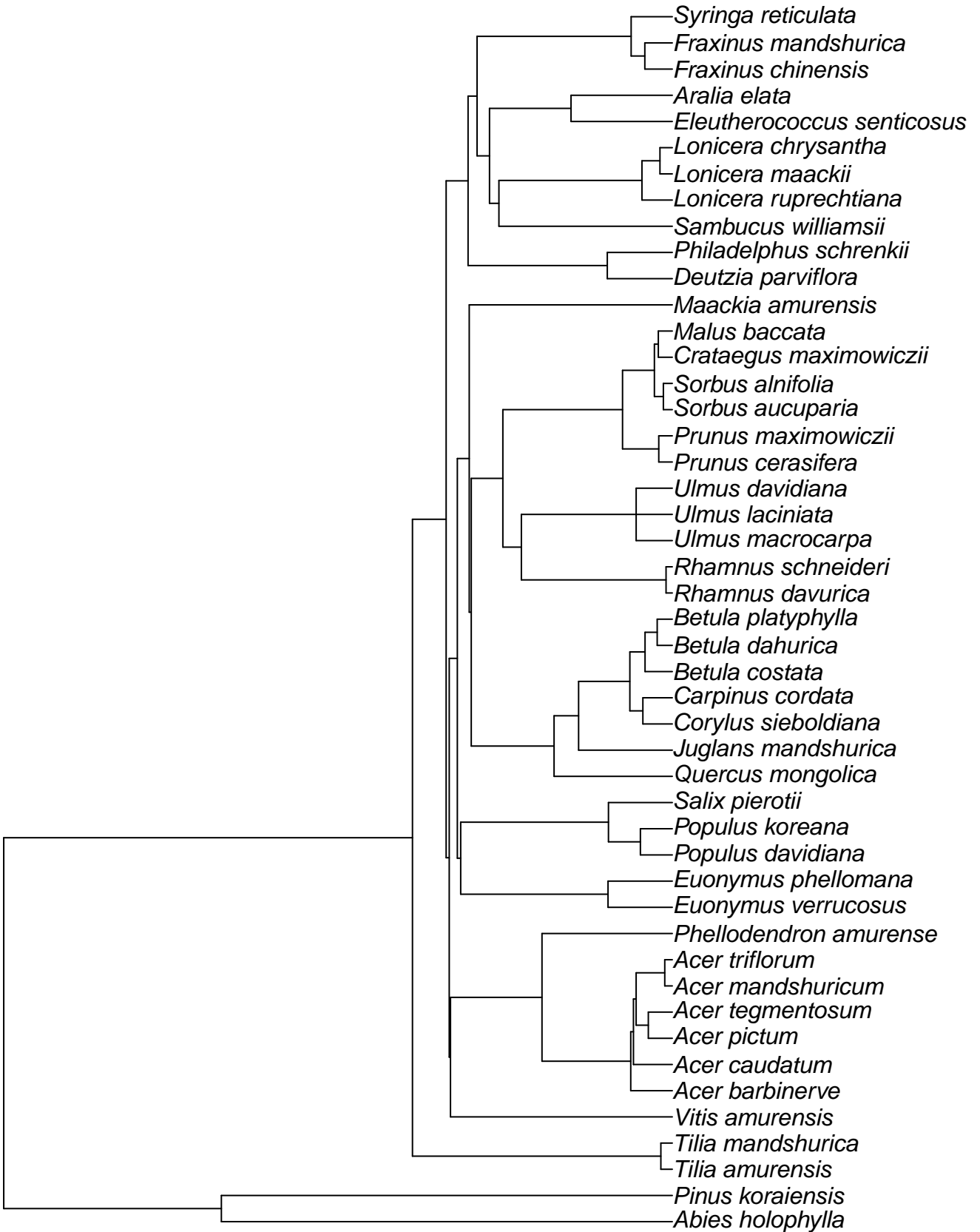

Supplement: Supplementary file 2 — Additional file 2. The phylogenies for Jiaohe plot. [file 12898_2017_143_MOESM2_ESM.pdf]

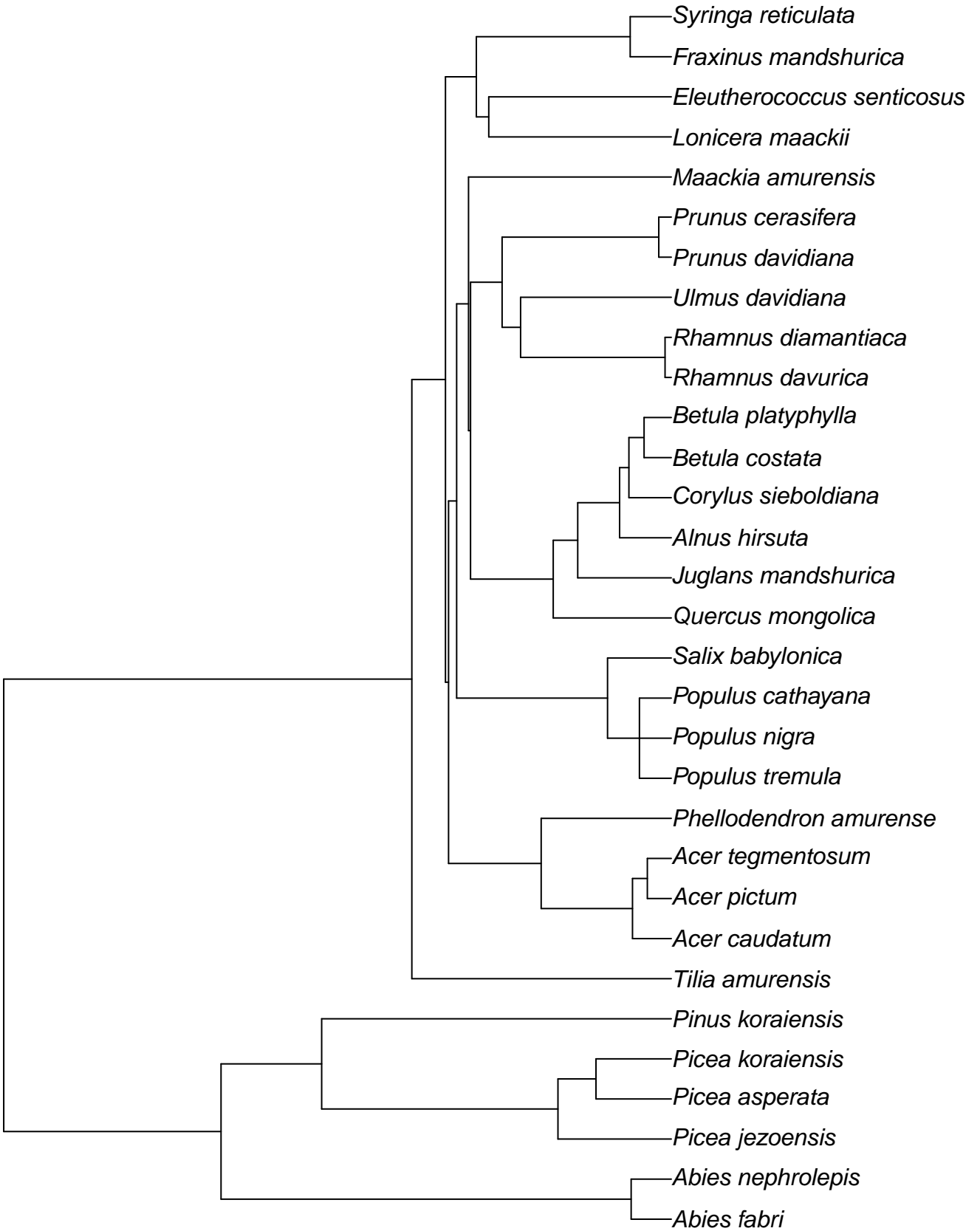

Supplement: Supplementary file 3 — Additional file 3. The phylogenies for Liangshui plot. [file 12898_2017_143_MOESM3_ESM.pdf]
